# Coordination of *Candida albicans* Invasion and Infection Functions by Phosphoglycerol Phosphatase Rhr2

**DOI:** 10.3390/pathogens4030573

**Published:** 2015-07-24

**Authors:** Jigar V. Desai, Shaoji Cheng, Tammy Ying, M. Hong Nguyen, Cornelius J. Clancy, Frederick Lanni, Aaron P. Mitchell

**Affiliations:** 1 Department of Biological Sciences, Carnegie Mellon University, 4400 Fifth Ave (MI- 289), Pittsburgh, PA 15213, USA; E-Mails: jigarkumar.desai@nih.gov or jigarvdesai@gmail.com (J.V.D.); tammythinks@gmail.com (T.Y.); 2 Department of Medicine, Infectious Disease Division, University of Pittsburgh, 3550 Terrace Street, Scaife S869, Pittsburgh, PA 15261, USA; E-Mail: sjc43@pitt.edu; 3 Department of Medicine, Infectious Disease Division, University of Pittsburgh, 3550 Fifth Ave, Suite 872 Scaife Hall, Pittsburgh, PA 15261, USA; E-Mail: mhn5@pitt.edu; 4 Department of Medicine, University of Pittsburgh, Pittsburgh, PA, USA; E-Mail: cjc76@pitt.edu; 5 Infectious Diseases Section, VA Pittsburgh Healthcare System, 3550 Fifth Ave, Suite 867 Scaife Hall, Pittsburgh, PA 15261, USA; 6 Department of Biological Sciences, Carnegie Mellon University, 4400 Fifth Ave (MI- 294C), Pittsburgh, PA 15213, USA; E-Mail: lanni@andrew.cmu.edu; 7 Department of Biological Sciences, Carnegie Mellon University, 4400 Fifth Ave (MI- 200B), Pittsburgh, PA 15213, USA

**Keywords:** *Candida albicans*, adherence, invasion, biofilm, intra-abdominal candidiasis, glycerol, turgor, infection, adhesin

## Abstract

The *Candida albicans RHR2* gene, which specifies a glycerol biosynthetic enzyme, is required for biofilm formation *in vitro* and *in vivo*. Prior studies indicate that *RHR2* is ultimately required for expression of adhesin genes, such as *ALS1.* In fact, *RHR2* is unnecessary for biofilm formation when *ALS1* is overexpressed from an *RHR2-*independent promoter*.* Here, we describe two additional biological processes that depend upon *RHR2*: invasion into an abiotic substrate and pathogenicity in an abdominal infection model. We report here that abiotic substrate invasion occurs concomitantly with biofilm formation, and a screen of transcription factor mutants indicates that biofilm and hyphal formation ability correlates with invasion ability. However, analysis presented here of the *rhr2Δ/Δ* mutant separates biofilm formation and invasion. We found that an *rhr2Δ/Δ* mutant forms a biofilm upon overexpression of the adhesin gene *ALS1* or the transcription factor genes *BRG1* or *UME6*. However, the biofilm-forming strains do not invade the substrate. These results indicate that *RHR2* has an adhesin-independent role in substrate invasion, and mathematical modeling argues that *RHR2* is required to generate turgor. Previous studies have shown that abdominal infection by *C. albicans* has two aspects: infection of abdominal organs and persistence in abscesses. We report here that an *rhr2Δ/Δ* mutant is defective in both of these infection phenotypes. We find here that overexpression of *ALS1* in the mutant restores infection of organs, but does not improve persistence in abscesses. Therefore, *RHR2* has an adhesin-independent role in abdominal infection, just as it does in substrate invasion. This report suggests that *RHR2*, through glycerol synthesis, coordinates adherence with host- or substrate-interaction activities that enable proliferation of the *C. albicans* population.

## 1. Introduction

Infection often begins with adherence. Microbes attach to host cells, tissues, or implanted devices, and then engage in pathogenic interactions such as invasion or inflammation. For many infectious microbes, the mechanisms that coordinate attachment and pathogenesis are well characterized. For example, in the case of enteropathogenic and enterohaemorrhagic *Escherichia coli*, the injected bacterial Tir protein mediates both bacterial attachment and host cytoskeletal rearrangements that ultimately lead to pathological effects [1]. In the case of *Helicobacter pylori,* contact with gastric epithelial cells induces formation of pili that translocate pathogenic effectors into host cells [2]. The pathogens *Yersinia pestis* and *Candida albicans* express invasin proteins that promote both attachment to and entry into host cells [3,4]. Because mechanisms that coordinate adherence and attachment are linchpins of infection, they provide insight into pathogenicity functions, and also present potential therapeutic targets.

Our focus is the fungus *C. albicans,* which is responsible for diverse mucosal and disseminated infections [5]. This organism can grow as ovoid yeast cells, cylindrical hyphal cells, and other cell types [6,7]. Hyphae express numerous surface adhesins that mediate adherence to host cells and abiotic surfaces [8,9,10]. Adherence thus establishes a foothold for invasion and biofilm formation. Hyphae from biofilms, on either mucosal or abiotic substrates, invade the underlying surface [11]. There are two modes of hyphal invasion. The first mechanism depends upon host cell functions: *C. albicans* hyphae induce their own endocytosis via the surface invasins Als3 and Ssa1 [9,12,13]. These invasins stimulate the actin-mediated endocytic pathway through interaction with E-cadherin and EGFR/HER2 on the epithelial surface [13,14]. The second mechanism is host cell-independent: *C. albicans* hyphae may invade a cell or substrate through exertion of force. This mechanism has been revealed through analysis of hyphal growth behaviors on abiotic surfaces [11,15]. Force-mediated invasion has been well studied in the plant pathogenic fungi *Magnaporthe oryzae* and *Colletotrichum graminicola* [16,17,18,19]. These fungi form a special melanized cell structure, the appresorium, which initiates invasion after adherence to the host surface. The appresorium generates enormous turgor via glycerol accumulation that drives a penetration peg through an underlying leaf surface [16]. It seems reasonable that *C. albicans* might also rely upon glycerol accumulation to generate turgor.

Glycerol has a prominent role in *C. albicans* biofilm formation, as first shown by Bonhomme and colleagues [20]. They found that the glycerol biosynthetic gene *RHR2*, which specifies 3-phosphoglycerol phosphatase, is up-regulated in biofilm cells compared to planktonic cells. This gene expression relationship reflects function, because they found that an *rhr2Δ/Δ* deletion mutant is defective in biofilm formation *in vitro*. Direct measurements have confirmed that *C. albicans* biofilm cells accumulate higher levels of glycerol than planktonic cells [21,22]. We confirmed the studies of Bonhomme and colleagues, and extended the observations to show that *RHR2* is required for biofilm formation *in vivo* in a catheter infection model [21]. The functional role of *RHR2* was unexpected, though: we observed that an *rhr2Δ* mutant has reduced adherence to a silicone substrate, and that it is defective in expression of biofilm adhesin genes [21]. Expression of adhesin genes such as *ALS1* from an *RHR2*-independent promoter permitted biofilm formation of an *rhr2Δ/Δ* mutant *in vitro* and *in vivo*. This observation indicated that the major role of *RHR2* in biofilm formation is to promote adhesin gene expression.

Here we have explored the function of *RHR2* under two other conditions: an *in vitro* surface invasion model, and an *in vivo* intra-abdominal candidiasis (IAC) model. We observe in both systems that *RHR2* is required for the wild-type biological activity. In addition, our observations support the idea that adhesin expression contributes to each phenotype. However, we find that *RHR2* is required for biological activity through mechanisms that are independent of adhesin expression as well. Our findings are consistent with the study by Wachtler *et al.* [23], who showed that *RHR2* (called *GPP1* in that study) is required for adherence-independent pathogenic interactions. We suggest that *RHR2* and glycerol accumulation serve to coordinate adhesion with other infection-related processes.

## 2. Results

### 2.1. Mechanical and Genetic Determinants of Substrate Invasion

**Figure 1:**
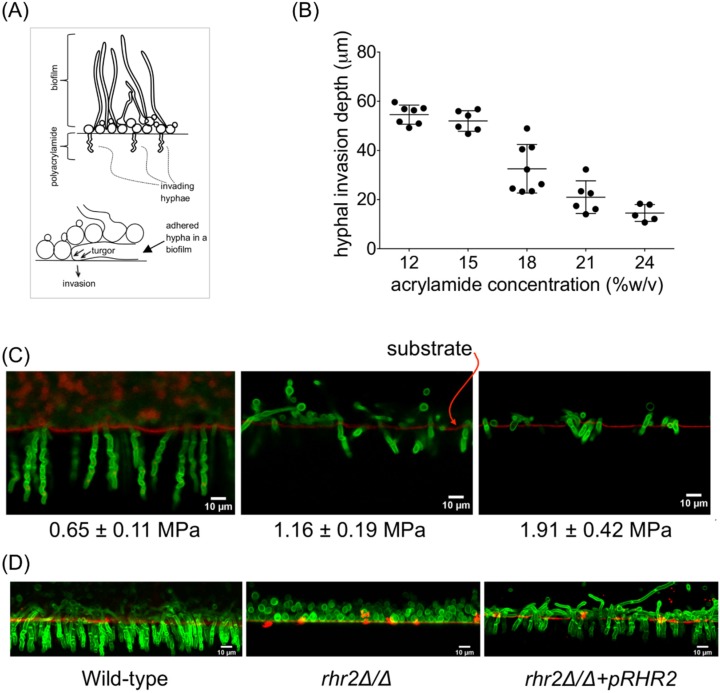
*Candida albicans* invasion into an elastic substrate. (**A**) Schematic diagram of invasion assay. Fungal cells grow as a biofilm on the surface, and invading hyphae penetrate into the polyacrylamide substrate. Our observations argue that turgor is required for invasion; (**B**) Depth of hyphal invasion *vs.* acrylamide concentration in polymerized gel substrate. Hyphal invasion depths were measured from collected images. Each point on the graph indicates an independent measurement. Error-bars correspond to standard deviation around the mean, which is marked by the horizontal line; (**C**) Confocal micrographs of *C. albicans* invasive growth into elastic hydrogels of increasing modulus of elasticity (G_polyacryl._). Circular polyacrylamide discs containing *C. albicans* biofilms were fixed, stained with Calcofluor white, and incubated with FluoSpheres to mark the surface. A 1–2 mm strip was then cut across the disc diameter, and rotated 90 degrees onto a cover-glass. Images were taken with 405 and 488 nm laser lines, then compiled and pseudo-colored green (Calcofluor white) and red (FluoSpheres). Numbers at bottom indicate G_polyacryl_ for each hydrogel; (**D**) Relationship between *RHR2* and invasion. Biofilms of the indicated strains were grown on polyacrylamide (18%/0.5% acryl./bis.), and specimens were prepared for imaging as described above. We note that *rhr2Δ/Δ* mutants are not defective in formation of hyphae [20]. The abundant yeast form cells in images of the mutant invasion assays represent yeast cells that sloughed off of the hydrogel surface and accumulated on the cover slip when the gel slice was rotated for imaging.

We developed a model for *C. albicans* invasion of noncellular substrates that employed polyacrylamide hydrogel discs. Cells were initially inoculated on the surface and incubated at 37 °C in YPD+serum medium. At 48 h there was confluent biofilm growth, and the hypothesis that we explore below is that biofilm formation is necessary for invasion. We quantified the depth of cell invasion into the substrate through microscopy (Figure 1A). The gel surface was marked with fluorescent microparticles, and fungal chitin was stained with Calcofluor White. We then measured the maximum distance over which chitin was detected beneath the surface through fluorescence microscopy of a slice through the hydrogel.

We observed that substrate invasion was accomplished by hyphal cells, in keeping with observations from many other *C. albicans* invasion models [24,25,26]. In addition, the invasion depth was inversely proportional to the acrylamide concentration in the substrate polymerization mixture (Figure 1B). Increases in the acrylamide concentration ultimately increase the rigidity of the substrate, an expectation that we verified by determination of the Young’s modulus of each hydrogel (Figure S1A,B). Our data are consistent with a model (see Discussion and Supplemental Information) in which invasive growth of the hydrogel depends on the effective hyphal turgor.

It seemed reasonable that biofilm formation on the surface of the substrate may be necessary for subsequent invasive growth. Our reasoning was that adhesion between the biofilm and the substrate would be necessary for mechanical penetration, much as has been found for the appressoria of plant pathogens [27]. If that is the case, then biofilm-defective mutants should be defective in invasive growth. We tested this prediction through assays of invasive growth capacities among a panel of 150 *C. albicans* transcription factor mutant strains [28]. Six mutants were defective in invasion (Table 1). Included in this group were five mutants known to have defects in both biofilm formation and hyphal morphogenesis. These mutants had individual deletions of the genes *BRG1*, *NDT80*, *EFG1*, *TEC1*, and *ROB1* [29,30,31,32]. We discovered an additional mutant, *dbp4Δ/Δ,* that was defective in invasion. (Although Dbp4 is not currently annotated as a transcription factor, it was included in the mutant collection [28].) The *dbp4Δ/Δ* mutant was also defective in biofilm formation under our conditions. These studies are consistent with the model that either or both biofilm formation and hyphal morphogenesis are required for substrate invasion.

**Table 1:** Transcription factor genes that are required for hyphal invasion.

Gene	Functional Description Form the *Candida* Genome Database	Mutant Phenotype
*BRG1*	Transcription factor; recruits Hda1 to hypha-specific promoters; Tn mutation affects filamentation; Hap43-repressed; Spider and flow model biofilm induced; required for Spider biofilm formation; Bcr1-repressed in RPMI a/a biofilms	defective in biofilm formation and invasive growth into polyacrylamide
*TEC1*	TEA/ATTS transcription factor; white cell pheromone response, hyphal gene regulation; required for Spider and RPMI biofilm formation; regulates BCR1; Cph2 regulated transcript; alkaline, rat catheter, Spider, flow model biofilm induced	
*NDT80*	Ortholog of Ndt80; meiosis-specific transcription factor; activator of CDR1 induction by antifungal drugs; required for wild-type drug resistance and for Spider biofilm formation; transcript induced by antifungal drug treatment	
*ROB1*	Zn(II)2Cys6 transcription factor; required for Spider model biofilm formation; mutant displays abnormal colony morphology and invasive growth; caspofungin repressed; flow model biofilm induced; rat catheter biofilm repressed	
*DPB4*	Putative DNA polymerase epsilon subunit D; null mutant is viable but slow-growing and displays abnormal invasive growth on SD and YPD media; Spider biofilm repressed	
*EFG1*	bHLH transcription factor; required for white-phase cell type, RPMI and Spider biofilm formation, hyphal growth, cell-wall gene regulation; roles in adhesion, virulence; Cph1 and Efg1 have role in host cytokine response; binds E-box	

### 2.2. Distinct RHR2 Requirements for Biofilm Formation and Invasion

Glycerol accumulation makes a significant contribution to turgor in many fungi [16,33]. The hypothesis that hyphal turgor is required for invasion under our conditions predicts that a mutant defective in glycerol accumulation will be defective in invasion. We tested that prediction with an *rhr2Δ/Δ* mutant, which is defective in the final step in glycerol synthesis. Previous studies have shown that glycerol accumulation is reduced two-fold during biofilm growth of this mutant [21,22]. We observed that the *rhr2Δ/Δ* mutant was severely defective in substrate invasion, and that integration of a wild-type copy of *RHR2* into the mutant genome restored invasion (Figure 1D). These results indicate that *RHR2* is required for invasion.

**Figure 2:**
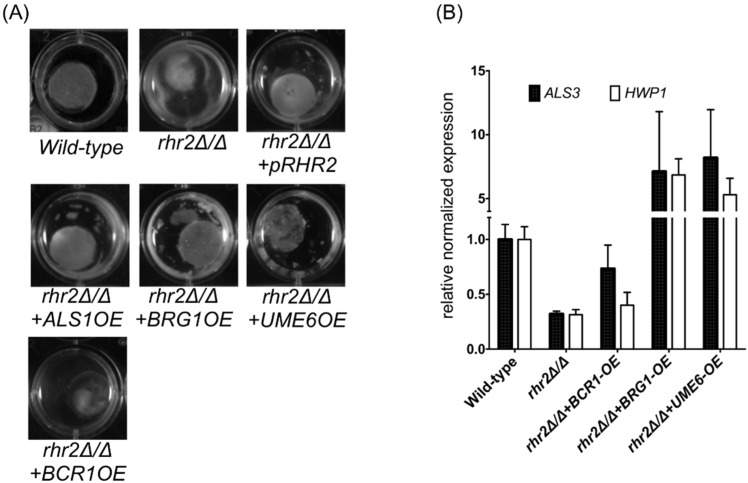

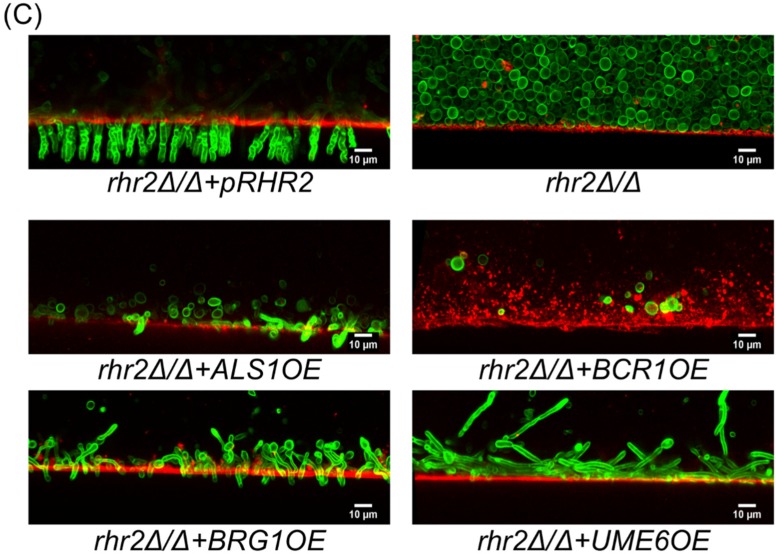
Functional requirement for *RHR2* in invasion. (**A**) Biofilm formation on polyacrylamide discs. Biofilms for the indicated strains were grown on polyacrylamide gel discs (18%/0.5% acryl./bis.) for 48 h, then photographed; (**B**) Gene expression analysis. The indicated strains were grown in YPD with 10% fetal bovine serum for the indicated strains for 8 h, and RNA was used for RT-qPCR measurements. *TDH3* expression was used as the normalization standard. The normalized data were then used to compute expression changes relative to the wild-type strain; (**C**) Confocal micrographs of invading hyphae. Biofilms of the indicated strains were grown on polyacrylamide gel discs (18%/0.5% acryl./bis.) and prepared for imaging as described in the Figure 1 legend. The strains used were: wild type (DAY185), JVD006 (*rhr2Δ/Δ + pRHR2*), JVD005 (*rhr2Δ/Δ*), JVD018 (*rhr2Δ/Δ + ALS1-OE*), JVD039 (*rhr2Δ/Δ + BCR1-OE*) and JVD051 (*rhr2Δ/Δ + UME6-OE*), and JVD065 (*rhr2Δ/Δ + BRG1-OE*).

Our prior studies revealed that expression of adhesin genes depends upon *RHR2* [21]. As a result, the *rhr2Δ/Δ* mutant is unable to form a biofilm, and overexpression of *ALS1* or other adhesin genes restores biofilm formation ability of the mutant. As expected, then, the *rhr2Δ/Δ* mutant was unable to form a biofilm in our invasion assay. The invasion defect of the *rhr2Δ/Δ* mutant might be a consequence of its biofilm defect. To test this hypothesis, we assayed invasion ability of an *rhr2Δ/Δ* mutant that overexpresses *ALS1.* We observed that *ALS1* overexpression restored biofilm formation under these conditions, but caused only a minor increase in invasion (Figure 2A,C). We further tested this hypothesis through overexpression of three transcriptional regulators of adhesin expression and biofilm formation in the *rhr2Δ/Δ* mutant background. Overexpression of two of these transcription factors, Brg1 and Ume6, caused increased expression of the adhesin genes *ALS3* and *HWP1*; the third transcription factor, Bcr1, had no such effect (Figure 2B). In addition, overexpression of Brg1 and Ume6, but not Bcr1, restored biofilm formation of the *rhr2Δ/Δ* mutant (Figure 2A). However, overexpression of any of these transcription factors had only a minor effect on invasion (Figure 2C). We conclude that *RHR2* is required for substrate invasion in some capacity beyond the expression of adhesin genes. We believe that this requirement reflects the contribution of glycerol accumulation to hyphal turgor, though our results do not rigorously exclude other possible roles for glycerol in this process.

### 2.3. RHR2 Function in Invasive Infection

**Figure 3:**
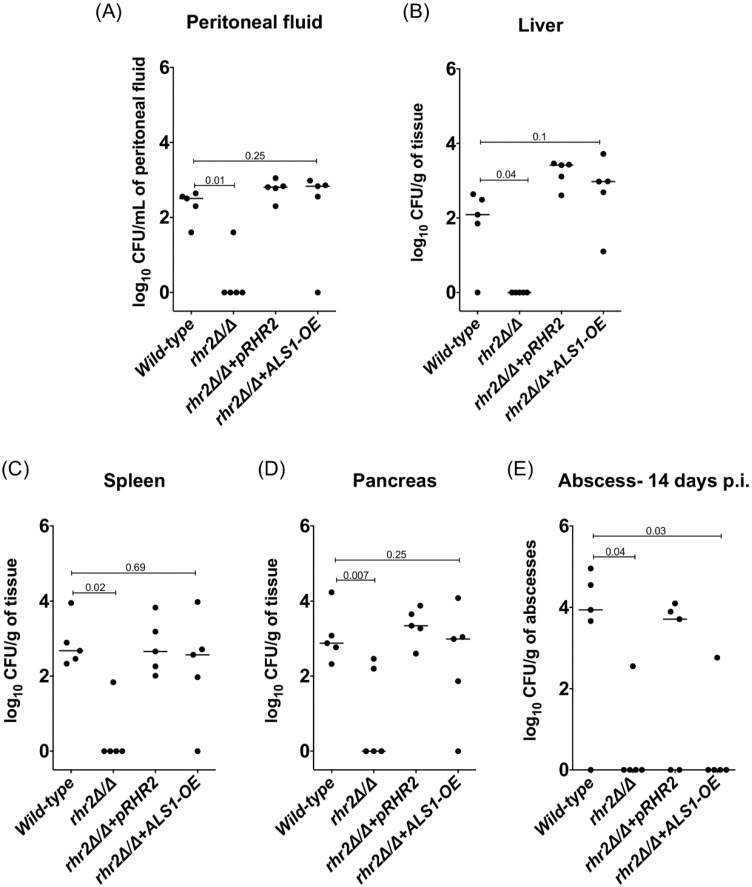
Association between *RHR2* function and abdominal infection. (**A**–**D**) Fungal burdens in peritoneal fluid and abdominal organs. Three days after infection, the peritoneum was lavaged twice with 1 mL PBS each, and the peritoneal fluid was obtained for CFU enumeration. The abdominal organs (liver, spleen and pancreas) were also removed, homogenized and enumerated for fungal burden. The plot shows median log_10_CFU /L of peritoneal fluid or log_10_CFU/g of tissue for the indicated *C. albicans* strains; (**E**) Fungal burden in intra-abdominal abscesses. Abscesses > 1 mm in size were excised and homogenized for CFU determination. The plot shows median log_10_CFU/g of abscesses for the indicated *C. albicans* strains. Note that very few abscesses were present at day 14 in the animals infected with the *rhr2Δ/Δ* and *rhr2Δ/Δ + ALS1-OE* strains. The strains used were wild type (DAY185), JVD006 (*rhr2Δ/Δ + pRHR2*), JVD005 (*rhr2Δ/Δ*), and JVD018 (*rhr2Δ/Δ + ALS1-OE*). The capped lines on each plot show values from Mann-Whitney tests.

We sought to determine whether the *in vitro* findings that point to two functions for *RHR2* might be relevant to infection biology. Prior studies indicate that biofilm formation in a rat catheter infection model depends mainly upon the adhesin expression function of *RHR2*. We turned to an intraabdominal candidiasis (IAC) model of infection because gene expression data suggested that glycerol synthesis is up-regulated in this model [34]. Indeed, the *rhr2Δ/Δ* mutant was severely defective in infecting the peritoneum and intra-abdominal organs (Figure 3A–D). For example, at three days post-infection, mutant titers were severely reduced in the peritoneum, liver, spleen and pancreas compared to the wild-type and complemented strains. In addition, the *rhr2Δ/Δ* mutant had reduced persistence within intra-abdominal abscesses compared to wild-type and complemented strains (Figure 3E and Figure S2). These results show that *RHR2* is required for infection of organs and persistence in abscesses in the IAC infection model.

These defects may arise from the *RHR2* function in adhesin expression. To test this hypothesis, we examined the *rhr2Δ/Δ* mutant that overexpresses *ALS1* in the IAC infection model (Figure 3A–E). *ALS1* overexpression restored the mutant’s ability to infect the peritoneum and organs. However, *ALS1* overexpression had no significant effect on persistence of the mutant within abscesses. These results argue that *RHR2* has two functions in the IAC infection model: it is required for adhesin expression in order to infect organs, and it is required in some capacity beyond the expression of adhesin genes that enables persistence in abscesses.

## 3. Discussion

The ability to invade tissues is a critical element in the arsenal of virulence traits of many microbial pathogens. Two invasion strategies are known to be employed by *C. albicans*. One is considered a passive mechanism in which *C. albicans* binds to host cell receptors to induce its endocytosis. The second is an active mechanism in which *C. albicans* can penetrate tissues and, rarely, implanted-abiotic substrates as well [11,15,35,36]. Here, we have focused on the active mechanism, with the specific goal of understanding the biological function of glycerol biosynthetic gene *RHR2.* Our study was motivated by two perplexing observations: that *RHR2* is very highly expressed during biofilm growth, and that *RHR2* is required for expression of several adhesin genes. Although *RHR2* is required for biofilm formation *in vitro* and *in vivo*, our studies indicated that this requirement reflected mainly the role of *RHR2* in adhesin gene expression [21]. One biological rationale for the coupling of *RHR2* and biofilm formation is that biofilm growth may generally precede substrate invasion. In that context, biofilm cells may express *RHR2* and accumulate glycerol in order to generate the turgor necessary for active invasion. The results in this study support that idea with two lines of evidence. First, we show that biofilm formation ability correlates with active invasion in a simple *in vitro* model. Second, we show that *RHR2* is required for invasion in some capacity beyond the control of adhesin gene expression. In addition, we find that *RHR2* is necessary for pathogenicity in an infection model, and that *RHR2* has two separable functions in that context as well.

Our observations argue that the two functions of *RHR2* in invasion *in vitro* are the stimulation of adhesin gene expression, and the generation of turgor. Adhesin expression enables biofilm formation, which occurs prior to invasion in our system. Our data suggest that turgor is required for invasion as well, based on a model in which hyphal invasion is represented as an expansion of a borehole in an infinite elastic medium (see Supplemental Information). According to the model, the extent of hyphal invasion into the substrate depends on the difference between hyphal turgor and the elastic modulus, or stiffness, of the substrate. The elastic modulus of polyacrylamide varies with the acrylamide concentration in the polymerization mixture, as confirmed by our direct measurements (Figure S1). The important observation is that hyphal invasion depth decreases linearly with increasing substrate elastic modulus. This relationship is expected if hyphal invasion is driven by hyphal turgor.

How much turgor is required for invasion? Theoretical extrapolation from hyphal invasion depth *vs.* polyacrylamide elastic moduli data (Figure 1B) suggests that hyphae can accumulate effective turgor of 0.7–1.0 MPa (7–-10 atm), comparable to what has been reported for single cells pressing against medical-grade silicone rubber, approximately ~1.2 MPa [37]. The fact that biofilm-derived hyphae have greater turgor than planktonic hyphae is consistent with the higher glycerol content of biofilm cells compared to planktonic cells [21,22].

Our studies also reveal that *RHR2* is required for infection in an IAC model. We observed previously that *RHR2* is dispensable for oropharyngeal candidiasis [21], and Wachtler *et al.* found that *RHR2* is required for a pathogenic interaction—specifically, damage—of oral epithelial cells [23]. The fact that these three infection models have different genetic requirements is consistent with our observation that gene expression profiles differ between the various infection environments [34,38]. Our results argue that one function of *RHR2* is to promote adhesin expression in this infection environment, because overexpression of *ALS1* suppresses one aspect of the *rhr2Δ/Δ* pathogenicity defect and enables organ infection. However, *ALS1* overexpression is not sufficient to permit persistence of the *rhr2Δ/Δ* mutant in abscesses. It is unlikely that *RHR2-*mediated turgor is required for tissue invasion, because the Young’s modulus of tissues is 1000-fold less than our hydrogels [39]. We suspect that the *RHR2-*dependence of persistence reflects the role of glycerol as an osmolyte, because abscesses present a hyper-osmotic environment [40]. It has been shown that *RHR2* is required for osmoadaptation of *C. albicans* [41,42]. *RHR2* is also required for adaptation to oxidative stress [43,44], which may be essential for persistence within abscesses as well. Therefore, the role of *RHR2* during IAC is complex, reflecting both adhesin regulation and additional functions.

Our studies suggest the hypothesis that *RHR2* functions to coordinate processes that are biologically, though not mechanistically, related. Under the two circumstances that we examined in this study, adherence provides a context in which additional *RHR2* functions are manifested. Such coordination functions are generally visualized for cell cycle or developmental regulators, in which a succession of biological steps is required to achieve an outcome. A major challenge now is to understand how the regulatory effects of *RHR2* and glycerol are relayed to mediate the various *RHR2* functions. Our data suggest one simple hypothesis: that the transcription factors Brg1 and Ume6 may mediate *RHR2* control over adhesin gene expression. This hypothesis is consistent with the observation that overexpression of either Brg1 or Ume6 caused increased adhesin expression in the *rhr2Δ/Δ* mutant. An alternative explanation is that Brg1 and Ume6 may act in parallel to a *RHR2-*responsive regulatory pathway. Future studies will distinguish between these models.

## 4. Experimental Section

### 4.1. Media and Strain Construction

*C. albicans* strains were grown on yeast extract-peptone-dextrose (YPD) medium. For biofilm assays, YPD with 10% fetal bovine serum (FBS) was used, a medium we refer to as YPD+serum. The overexpression strains were constructed as described previously [21]. All *C. albicans* strains are listed in Table 2.

**Table 2:** *C. albicans* strains used in this study.

Strain	Genotype	Source
DAY185 (Wild-type)	*ura3Δ::λiimm434 HIS1::his1::hisG ARG4::URA3::arg4::hisG*	[45]
	*ura3Δ::λiimm434 his1::hisG arg4::hisG*	
JVD005 (*rhr2Δ/Δ)*	*ura3Δ::λimm434 arg4::hisG his1::hisG::pHIS1 rhr2::ARG4*	[21]
	*ura3Δ::λimm434 arg4::hisG his1::hisG rhr2::URA3*	
JVD006 (*rhr2Δ/Δ + pRHR2)*	*ura3Δ::λimm434 arg4::hisG his1::hisG::pHIS1-RHR2 rhr2::ARG4*	[21]
	*ura3Δ::λimm434 arg4::hisG his1::hisG rhr2::URA3*	
JVD039 (*rhr2Δ/Δ + BCR1-OE)*	*ura3Δ::λimm434 arg4::hisG his1::hisG::pHIS1 rhr2::ARG4*	[21]
	* ALS1::pAgTEF1-NAT1-AgTEF1UTR-TDH3-BCR1*	
	*ura3Δ::λimm434 arg4::hisG his1::hisG rhr2::URA3 BCR1*	
JVD065 (*rhr2Δ/Δ + BRG1-OE)*	*ura3Δ::λimm434 arg4::hisG his1::hisG::pHIS1 rhr2::ARG4*	This study
	*ALS1::pAgTEF1-NAT1-AgTEF1UTR-TDH3-BRG1*	
	*ura3Δ::λimm434 arg4::hisG his1::hisG rhr2::URA3 BRG1*	
JVD051 (*rhr2Δ/Δ + UME6-OE)*	*ura3Δ::λimm434 arg4::hisG his1::hisG::pHIS1 rhr2::ARG4 *	This study
	*ALS1::pAgTEF1-NAT1-AgTEF1UTR-TDH3-UME6*	
	*ura3Δ::λimm434 arg4::hisG his1::hisG rhr2::URA3 UME6*	
JVD018 (*rhr2Δ/Δ + ALS1-OE)*	*ura3Δ::λimm434 arg4::hisG his1::hisG::pHIS1 rhr2::ARG4 *	[21]
	*ALS1::pAgTEF1-NAT1-AgTEF1UTR-TDH3-ALS1*	
	*ura3Δ::λimm434 arg4::hisG his1::hisG rhr2::URA3 ALS1*	

The transcription factor mutant library used in this study has been described by Homann, O.R. *et al.*, 2009 [28].

### 4.2. RNA Sample Preparation

For gene expression analysis, the strains (wild-type-DAY185, *rhr2Δ/Δ*-JVD005, and *rhr2Δ/Δ + pRHR2*-JVD006, *rhr2Δ/Δ + BCR1OE*-JVD039, *rhr2Δ/Δ + UME6OE*-JVD051 and *rhr2Δ/Δ + BRG1OE*-JVD065) were grown overnight in YPD medium at 30 °C. Cells from overnight cultures in YPD medium were added to 50 mL YPD + 10%FBS at a final OD_600nm_ of 0.2. Cells were grown for additional 8 h at 37 °C with 225 rpm agitation in the incubator. The cells were harvested by filtering the cell suspension on a vacuum manifold. The filters were flash frozen immediately after harvesting each sample. The cells were kept frozen on filters at −80 °C until RNA extraction. RNA was extracted as described previously [21]. Briefly, cells were re-suspended from filters with 1.5 mL ice-cold distilled water, followed by 15 to 30 s of vigorous vortexing. The re-suspended cells were transferred to a 1.5-mL tube and spun down according to the manufacturer’s protocol. During the cell disruption step, the cells were beaten with a Next Advance Bullet Blender for 3 min at 4 °C for cell lysis. Post extraction procedure, the RNA was stored −80 °C until further use.

### 4.3. Quantitative RT PCR

10 µg RNA was rendered DNA-free using a kit (Ambion). The DNA-free RNA was then used to synthesize cDNA using the AffinityScript multiple temperature cDNA synthesis kit (Stratagene). A control reaction was included, in which reverse transcriptase was omitted to ensure the absence of DNA contamination. 2×iQ SYBR Green Supermix (Bio-Rad), 1 µL of first-strand cDNA reaction mixture, and 0.1 µM of primers were mixed in a total volume of 25 µL per reaction. Real-time PCR was performed in triplicate using a CFX Connect Real-Time System (Bio-Rad). The program for amplification had an initial denaturation step at 95 °C for 5 min, followed by 40 cycles of 95 °C for 45 s and 58 °C for 30 s. Product amplification was detected using SYBR Green fluorescence at the end of the 58 °C step. Gene expression was determined using Bio-Rad iQ5 software (ΔΔCT method), with *TDH3* and 16S RNA expression used for normalization.

### 4.4. Polyacrylamide Gels for Invasion Assay

Stock solutions of acrylamide (Bio-Rad) and bisacrylamide (Bio-Rad) were prepared at concentrations of 35% and 2.5%w/v, respectively. The required volumes were withdrawn and mixed together. Gelatin powder (Sigma) was added at a final concentration of 0.1%w/v to the water, required to make up the final volume. Gelatin was dissolved by heating the gelatin-water suspension in a microwave for ~5–10 s. The gelatin solution was then added to the acrylamide-bisacrylamide mixture. To this mixture of acrylamide-bisacrylamide and gelatin, 10×PBS was added such that the final concentration of PBS is 1×. Thus prepared mixture in 1×PBS was then transferred to a petri dish for degassing. Degassing under vacuum was performed in a standard degasing chamber for 30 min. After degassing, the polymerization catalysts: TEMED (tetramethylethylenediamine) and APS (ammonium persulfate) were added at concentrations of 0.05%w/v each. The petri dish was swirled gently to mix the contents uniformly. Immediately, the unpolymerized mixture was poured between two glass plates. The glass plates yielding 1.5 mm thick gels were utilized. A layer of water-saturated butanol was applied by putting ~500 μL atop the gel. Polymerization was continued for 30 min–1 h. The polymerized gels were removed from the molds and washed with distilled water by swirling at low speed on an orbital shaker (5×). These were later trimmed for either biofilm growth or elasticity analysis.

### 4.5. Elastic Modulus Measurement

For elastic moduli measurement, gel strips were cut to 10 mm width with a sharp razor. The cut strip was then glued to rectangular pieces of glass slides at both of its ends along the long axis, using Superglue (cyanoacrylate cement). With the help of paper clips, the gel strip with the glass slides was suspended under gravity. Tensile stress was applied at the free end of the suspending gel-strip by hanging weights from the lower piece of glass. The strain was then measured by recording a change in gel extension in response to two different weights: m_1_ and m_2_. The initial length L_1_ was recorded with a digital Vernier caliper when the weight m_1_ was suspended to stretch the gel. The final length L_2_ was recorded the same way when m_2_ was suspended to stretch the gel. The strain was calculated as the normalized difference: (L_2_−L_1_)/L_1_. At-least three strain values were determined for at least three different weights. The resulting data along with the gel cross-sectional area were then used to plot stress-strain curves. The ratio stress/strain is Young’s modulus (Figure S1B).

### 4.6. Confocal Imaging of Invasion

The strains under analysis were grown overnight in YPD at 30 °C. The overnight cultures were used to inoculate 2 mL YPD+10%FBS at OD_600nm_ of 0.5 in wells, containing polyacrylamide discs pretreated overnight with fetal bovine serum (FBS). The cells were adhered to polyacrylamide by incubating for 90 min in an incubator-shaker at 60 rpm and 37 °C. After 90 min the cells not adhering to substrate were washed off with phosphate buffer saline (PBS) and fresh YPD + 10%FBS was then placed in each well. After an incubation for 48 h at 37 °C with 60 rpm agitation, the medium was aspirated out and the circular polyacrylamide discs, containing C. albicans biofilms, were fixed with 4% formaldehyde (30 min) and subsequently co-incubated with 0.185 mg/mL Calcofluor white and 0.05% green FluoSpheres in 1×PBS (1 h). Roughly 1–2 mm sized strips were then cut across the disc diameter, with a sharp razor and the strip was laid over on its side on a cover-glass, housed within a culture dish. Serial stacks of images were acquired with a Zeiss LSM 510 Meta/DuoScan inverted spectral confocal microscope using a 40×/1.2NA-water immersion objective with the laser lines at 405 nm and 488 nm. The Zen 2009 software was used to obtain the desired Z-stack images. The serial image stacks were processed in FIJI (http://rsbweb.nih.gov/ij/). The apical view projections were computed from the intensity corrected image stacks.

For hyphal invasion depth measurement, we used the standard line measurement tool in FIJI. For 5–8 replicates, the depth was measured between the FluoSphere marked polyacrylamide surface and the longest hypha that invaded into the substrate. We performed the measurements in similar manner for the polyacrylamide discs of different elastic moduli.

### 4.7. Murine Model of Intra-Abdominal Candidiasis (IAC)

Animal experiments were performed according to the University of Pittsburgh Institutional Animal Care and Use Committee guidelines as described previously [34]. Mice were infected intra-peritoneally (IP) with 100 μL of 1 × 10^6^ CFU *C. albicans* intermixed with steam-sterilized mouse feces. Mouse feces were homogenized in a tissue grinder, suspended in normal saline (NS) to form a 5% wt/vol mixture, and sterilized in a steam autoclave (15 min, 2 bar, 120 °C). Non-viable organisms were confirmed by culture in Luria Broth (for aerobic bacteria), chopped meat broth (for anaerobic bacteria) and YPD media (for fungi). To assess for peritonitis, the mice were sacrificed on day 3 post-infection. The peritoneum was then washed two times with 1 mL PBS, and the lavage fluid underwent *Candida* enumeration. The liver, spleen and pancreas were harvested for tissue burden enumeration. To assess for tissue burden within abscesses, the mice were sacrificed 14 days after infection, and abdominal cavities explored. Abscesses >1 mm in diameter were excised from surrounding tissues, homogenized and enumerated for fungal burden. Tissue burdens are presented as log_10_CFU/L of peritoneal fluid or log_10_CFU/gram of tissue. The difference in tissue burdens between mice infected with different strains was determined by Mann-Whitney test. A value <0.05 was considered statistically significant.

## 5. Conclusions

The glycerol biosynthetic gene *RHR2* and, by inference, glycerol have multiple biological functions in the fungal pathogen *C. albicans*. Prior studies showed that *RHR2* is required for expression of *ALS1* and other adhesin genes. Our studies here of surface invasion *in vitro*, and of a mouse intra-abdominal candidiasis model, reveal that adhesin expression does not account for all *RHR2* functions in invasion and pathogenesis. Our results argue that turgor generation is a second critical biological function of *RHR2* and glycerol.

## Supplementary Information

Supplementary materials can be accessed at: http://www.mdpi.com/2076-0817/4/3/573/s1.
