# Supplementary material for: Coordination of Candida albicans Invasion and Infection Functions by Phosphoglycerol Phosphatase Rhr2
Source: Pathogens. 2015 Jul 24;4(3):573–89. doi: 10.3390/pathogens4030573 (PMC4584273; doi:10.3390/pathogens4030573)
Supplement: Supplementary file 1 [file pathogens-04-00573-s001.pdf]

Pathogens **2015**, *4*, 573-589; doi:10.3390/pathogens4030573

OPEN ACCESS

pathogens

ISSN 2076-0817

www.mdpi.com/journal/pathogens

Supplemental Information

Coordination of *Candida Albicans* Invasion and Infection Functions by Phosphoglycerol Phosphatase Rhr2

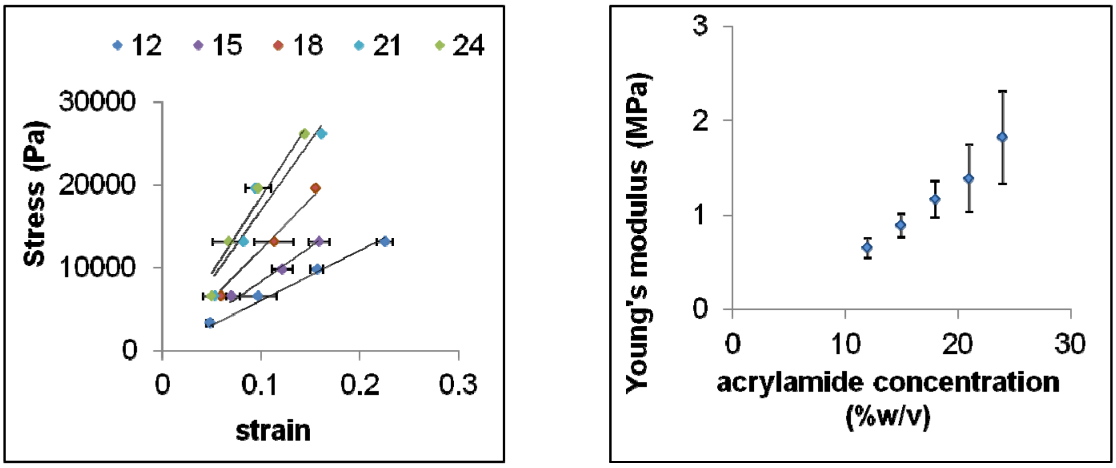

(A) (B)

Figure S1. (**A**) Stress-strain curves for the four different polyacrylamide hydrogel substrate. Four different hydrogels were polymerized where acrylamide was used at 12%, 18%, 21% and 24% w/v while bisacrylamide was used at a constant concentration of 0.5 w/v. The legend on right shows markers corresponding to different hydrogels made by using different % of acrylamide. The horizontal error-bars mark standard deviation in strain values. (**B**) Young's moduli for the four different hydrogels. Young's moduli were computed as the stress/strain ratios from the data displayed in Figure 1(A). The error-bars mark standard deviation in moduli values.

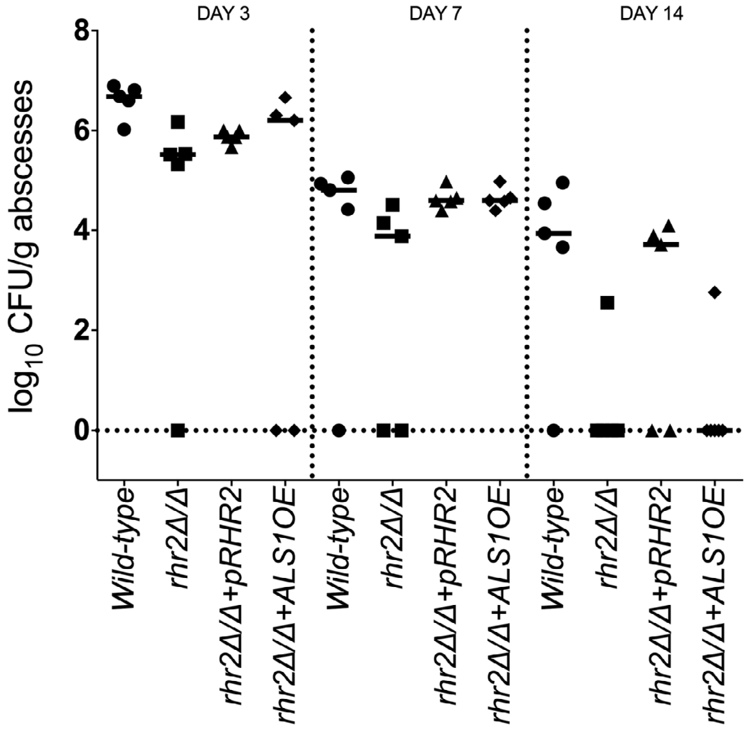

Figure S2. Fungal burden in intra-abdominal abscesses. Abscesses > 1 mm in size were used for CFU determination. The plot shows logCFU/g of abscesses for the indicated *C. albicans* strains for abscesses that were harvested 3, 7 and 14 days after infection.

Expansion of a Cylindrical Tube or Borehole in an Elastic Medium under Internal Pressure

A long cylindrical borehole or tube in a concentric elastic cylinder or an infinite elastic solid can be represented in the **relaxed state** (P = 0) by concentric cylindrical shells in which the innermost shell has internal radius R_0, and external radius R_1 (Figure S3). Upon pressurization of the borehole, the inner radius grows to r_0 and the external radius grows to r_1, with purely radial displacement (U) of every material point ($R \geq R_0$, $r \geq R$). If the material is incompressible, U(R) is completely defined by geometry:

$$U(R) = r(R) - R = (R^2 + r_0^2 - R_0^2)^{1/2} - R \quad (1)$$

U(R) is greatest at the inner radius, where it equals $r_0 - R_0$, and decreases asymptotically as 1/R.

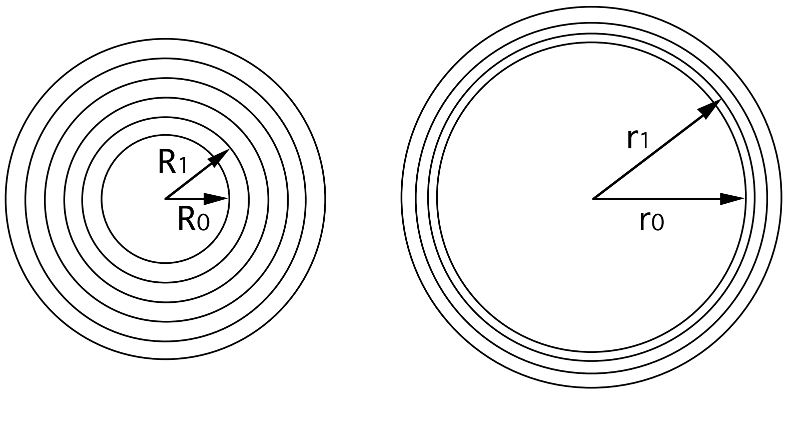

Figure S3. Elastic medium in relaxed state (left) P = 0, and in expanded state (right) P > 0. In the relaxed state, R_0 is the inner radius of the tube.

In the simplest case of an incompressible rubber-like material, Treloar [1–3] derived the **principal stresses** in terms of the local principal strain ratios. In the case of expansion of a cylindrical tube, it can be shown that the three Treloar principal stresses are the diagonal elements of the stress tensor in which the three principal directions are radial, circumferential, and axial. For the **infinite elastic solid**, the three principal stresses are:

$$\sigma_{11\infty} = -G + G\,R^2/r^2 = -G\,(1 - R^2/r^2) \qquad \text{(radial)} \qquad (2)$$

$$\sigma_{22\infty} = -G + G\,r^2/R^2 = G\,(r^2/R^2 - 1) \qquad \text{(circumferential)} \qquad (3)$$

$$\sigma_{33\infty} = -G + G = 0 \qquad \text{(axial)} \qquad (4)$$

Radial stress is negative since $R < r$ for all $P > 0$. Circumferential stress is always positive, for the same reason. Surprisingly, pressurization of the tube causes no axial stress.

From the boundary condition of hydrostatic pressure P on the internal surface, the relation between R_0, r_0 and P is determined:

$$-P = -G + G\,R_0^2/r_0^2 \qquad (5)$$

Solving for r_0:

$$r_0 = [G/(G-P)]^{1/2}\,R_0 \qquad (6)$$

This result shows that, for any pressure less than the modulus of elasticity ($P < G$), a finite expansion will be obtained for a tube of any nonzero internal radius in the relaxed state. If $P \rightarrow G$, the expanded inner radius can be made arbitrarily large, implying that runaway inflation may occur. Practically, expansion would be halted by the elastic limit of the material, or freed by material failure. In principle, a "borehole" may be initiated even when $R_0 = 0$ by any mechanism that can generate $P = G$. Conversely, it is shown by Equation (5) that $P = G$ for ***any*** finite r_0, when $R_0 = 0$. Therefore, for invasion of an elastic solid, generation of internal pressure equal to the modulus ($P = G$) allows expansion from $r = 0$ to any internal radius, r_0. This is, of course, a reversible (equilibrium) result. Expansion or invasion at a finite rate would require $P > G$.

Invasion Progress after Tube Initiation by a Hyphus

If we consider the hyphus as a cylinder of uniform diameter which grows by extension of the tip cell after each mitosis, we can apply a model originally derived for plant cell growth [4–6] in which the cell wall has an elastic modulus E and a yield stress for irreversible plastic extension, P_c. If the hyphus is invading an elastic solid, it also must expand against the modulus G. As shown above, this requires a pressure $P = G$. Under this condition, the invasive growth rate depends on the turgor pressure in excess of $P_c + G$;

$$P_{eff} = P - (P_c + G) \qquad (7)$$

In the case of steady growth under non-nutrient-limited conditions (such as in our eperiments), we make the simplifying assumption that hyphal cells maintain a relatively constant osmolarity and resulting turgor pressure through the cell cycle. In this case, the volumetric cell growth equation developed by Lockhart [4] and extended by Ortega [5,6] can be simplified by dropping the elastic term:

$$(1/V)dV/dt = \phi P_{eff} \tag{8}$$

In the cell growth equation, ϕ is the linear cell wall extensibility above the yield stress. Since hyphal growth occurs essentially along one axis, (1/V)dV/dt = (1/L)dL/dt where L is the tip cell length:

$$dL/dt = \phi P_{eff} L \tag{9}$$

in which case cell elongation is exponential:

$$L(t) = L_0 \exp[\phi P_{eff} t] \qquad t \geq 0 \tag{10}$$

Our observations with invading hyphae show that the invading hyphae have septa, thus they have undergone mitosis. We assume that during invasion, the tip cell elongates, then stops, undergoes mitosis and septates to form a new tip cell of length L_0. The new tip cell then continues the growth process. Under our tacit assumption, all the cells in a hyphal chain, except the one at the tip, go to a G_0 state. Hence, the growth curve for a single hyphal chain would contain a series of exponential "spurts", each of duration equal to the cell-cycle period. An overall plot of hyphal length *vs.* time would look similar to a series of exponential "spurts" arranged in tandem. The mean slope would represent the change in hyphal length per cell cycle period, t_{cc}, which is the mean speed of invasion (v_i):

$$v_i = \{L(t_{cc}) - L_0\}/t_{cc} \tag{11}$$

$$= L_0 \{ \exp[\phi P_{eff} t_{cc}] - 1\}/t_{cc} \tag{12}$$

which can be linearized under the growth conditions used in these experiments:

$$v_i \approx L_0 \phi P_{eff} = L_0 \phi (P - (P_c + G)) \tag{13}$$

In our invasion experiments, the **invasion depth** observed at time "t_g" (= 48 h) for the wild-type strain is very nearly equal to the embedded hyphal length:

$$L_h = v_i t_g = L_0 \phi (P - P_c - G) t_g \tag{14}$$

With a fixed growth period (t_g = 48 h), this relation is a linear equation in G, the external elastic modulus:

$$L_h = (L_0 \phi (P - P_c) t_g) - (L_0 \phi t_g) G \tag{15}$$

which shows that increasing the modulus G should decrease the observed invasion depth, L_h, and predicts that invasion will fail when $G = P - P_c$.

References

1. Atkin, R.J.; Fox, N. *An Introduction to the Theory of Elasticity*; Longman Group Ltd.: London, UK, 1980; pp. 72–75.
2. Treloar, L.R.G. The elasticity of a network of long-chain molecules. I. *Trans. Faraday Soc.* **1943**, *39*, 36–41.
3. Treloar, L.R.G. The elasticity of a network of long-chain molecules. II. *Trans. Faraday Soc.* **1943**, *39*, 241–246.
4. Lockhart, J.A. An analysis of irreversible plant cell elongation. *J. Theor. Biol.* **1965**, *8*, 264–275.

5. Ortega, J.K.E. Augmented growth equation for cell wall expansion. *Plant Physiol.* **1985**, *79*, 318–320.
6. Proseus, T.E.; Ortega, J.K.E.; Boyer, J.S. Separating growth from elastic deformation during cell enlargement. *Plant Physiol.* **1999**, *119*, 775–784.

© 2015 by the authors; licensee MDPI, Basel, Switzerland. This article is an open access article distributed under the terms and conditions of the Creative Commons Attribution license (http://creativecommons.org/licenses/by/4.0/).
